# Study on High-Ductility Geopolymer Concrete: The Influence of Oven Heat Curing Conditions on Mechanical Properties and Microstructural Development

**DOI:** 10.3390/ma17164011

**Published:** 2024-08-12

**Authors:** Ruihao Luo, Runan Liu, Guang Qin, Minyang Jiang, Yixian Wu, Yongchang Guo

**Affiliations:** 1 School of Civil and Transportation Engineering, Guangdong University of Technology, Guangzhou 510006, China; luoryann@outlook.com (R.L.); 2112209125@mail2.gdut.edu.cn (R.L.); 2 Foshan Road and Bridge Supervision Station Corporation Limited, Foshan 528313, China; qinguang1984@163.com; 3 Fujian Xingyuan Construction Engineering Development Corporation Limited, Fuzhou 350001, China; fjxyjsgc@fjxyjsgc.cn; 4 Fujian Chengshuo Construction Engineering Corporation Limited, Fuzhou 350800, China; rang1128@126.com

**Keywords:** high-ductility geopolymer concrete, oven heat curing, curing duration, mechanical properties, bridging effect

## Abstract

Low carbon and high performance have become key trends in the development of construction materials. Understanding the mechanism by which curing conditions affect the mechanical properties of high-ductility geopolymer concrete (HDGC) is of significant importance. This study investigated three sealing curing temperatures (room temperature, 45 °C, and 60 °C) and four curing durations (1 day, 3 days, 5 days, and 7 days), while considering two final curing ages (7 days and 28 days) to explore their effects on the axial tensile and compressive properties of HDGC. The results showed that both 45 °C and 60 °C could improve the brittle failure of HDGC under axial compressive loading. However, curing at 60 °C and for durations longer than 1 day in an oven would catalyze the formation of eight-faced zeolite crystals within the slag–fly ash geopolymer matrix, and it could weaken the matrix’s pore structure and subsequently affect the material’s later strength development. Nevertheless, oven heat curing enhanced the bridging effect between the fibers and the matrix, partially compensating for the reduction in the initial tensile strength of HDGC. This follows the pseudo-strain-hardening material’s saturation cracking criterion to enhance the strain-hardening behavior of HDGC and improve its tensile energy absorption capacity. A curing condition of 45 °C for 5 days is recommended to maximize the ductility of HDGC. This study provides important theoretical support for the design and promotion of green, low-carbon, high-ductility composite materials.

## 1. Introduction

In the modern construction industry, the development of high-performance materials and the overall reduction in carbon emissions have become hotspots. Traditional concrete materials are gradually revealing their limitations in durability, strength, and environmental friendliness [1,2,3,4]. Although Engineered Cementitious Composites (ECCs) are considered highly promising due to their high ductility and toughness, their high demand for cement does not align with the construction industry’s pursuit of low-carbon and environmentally friendly development [5,6,7,8,9,10,11]. Geopolymer alkali-activated materials, typically produced by activating aluminosilicates with alkaline activators through a geopolymerization process that involves dissolution, rearrangement, condensation, and solidification [12,13,14,15], are now considered excellent alternatives to Ordinary Portland Cement (OPC). The use of geopolymer alkali-activated materials in concrete is associated with a lower carbon footprint, excellent thermal resistance, and durability [1,13,14,16]. Ohno et al. designed Engineered Geopolymer Composites (EGCs) based on the ECC design methodology, resulting in the creation of environmentally friendly high-ductility composite materials [17].

Existing research indicates that the mechanical properties of EGCs are influenced by various variables, including the type of precursor [18], particle size distribution of the materials [19], alkalinity and modulus of the alkali activator [20], water-to-binder ratio [21], and curing conditions [22,23,24]. Among these, curing conditions are one of the most critical factors in designing high-performance geopolymer composites, as they significantly affect the development of the geopolymer matrix and consequently impact the mechanical properties. In addition to standard water curing as per ASTM-C511 [25], environmental/room temperature curing (25 °C), oven heat curing, steam curing [26], and intermittent/cyclic heat curing [27] have been identified as catalytic methods that can influence the formation of geopolymer binders. Alexandre et al. [28] found that when the binder products are aluminosilicates, curing temperature and age significantly impact the material’s compressive strength. Additionally, the use of fibers and mineral materials is believed to reduce common microcracks in alkali-activated binder matrices. Mo et al. [29] pointed out that elevated curing temperatures could promote the dissolution of geopolymer precursor particles and accelerate the polymerization process. Prolonged curing times favor the early formation of a hardened structure in the matrix.

Changing the curing temperature is widely studied due to its ease of implementation and significant effects. Commonly used geopolymer precursors are typically cured at temperatures ranging from 40 °C to 80 °C for 4–48 h [30,31,32,33]. Al Bakri et al. [34] reported that oven heat curing accelerates the development of compressive strength in fly ash-based geopolymers. Similarly, Singh et al. [24] discussed the impact of curing temperature on the mechanical properties of fly ash-based geopolymers, noting that higher temperatures increase the dissolution rate of the glass phase in fly ash, allowing materials cured at 85 °C to achieve the same compressive strength in a shorter period as those cured at 40 °C. The optimal curing temperature for enhancing mechanical properties is not absolute and varies between different precursor compositions. Manvendra Verma [35] found that slag–fly ash-based geopolymer concrete significantly improved in compressive strength after being oven-cured at 80 °C for 24 h, with further gradual enhancement after 28 days compared to ambient-cured samples. Kürklü [36] reported that 60 °C oven curing yielded the highest compressive strength for slag-based geopolymers. Türker et al. [37] evaluated the effects of two curing regimes (ambient curing at 21 ± 2 °C and 60% humidity, and high-temperature curing at 60 °C for 6 h) on the high-temperature performance of alkali-activated mortars. The results indicated that high-temperature curing resulted in a notably high compressive strength, though the flexural strength significantly decreased. Additionally, compared to ambient curing and OPC mortars, high-temperature curing reduced mass loss under high-temperature conditions.

Oven heat curing duration also significantly impacts the mechanical properties of geopolymers. Existing studies often set the heat curing duration within 48 h, with the optimal curing duration defined as 6–24 h [26,36,38,39,40,41,42,43,44,45,46,47]. Guo et al. [48] found that the compressive strength of fly ash-based geopolymers cured at 75 °C began to decrease after 8 h due to matrix structure damage and dehydration shrinkage, attributing the decline primarily to prolonged curing. Rovnanik et al. [49] analyzed the mechanism by which prolonged curing led to pore accumulation, forming macropores in the matrix structure and negatively affecting the material’s mechanical properties. While extended heat curing has evident adverse effects on fly ash-based geopolymers, this also depends on the precursor composition. The most favorable curing conditions for slag–alkali-activated products are considered to be within a curing temperature range of 30 °C to 60 °C and a curing duration of 2 h to 14 days [50,51,52,53].

The superior tensile deformation capacity and tensile strength of EGC compared to OPC are largely attributed to the effective bridging performance between the fibers and the matrix, which is closely linked to the microstructure of the EGC matrix. Lv [54] and Idawati [55] observed similar phenomena, noting that some of the Ca released from slag dissolution is incorporated into N-A-S-H, forming N-(C)-A-S-H-type gels. Prolonged heat curing promotes the formation of more cross-linked binding products and a denser microstructure. Puertas [56] found that at 25 °C ambient curing, the alkali activation of slag was sufficient, with only a partial dissolution of fly ash participating in the reaction, resulting in the absence of “geopolymer gel” in the matrix. Fly ash requires temperatures between 40 °C and 85 °C to accelerate the reaction. S.K. Nath [57] compared the effects of curing conditions and alkali solution concentration on the microstructure and morphological evolution of alkali-activated fly ash, pointed that with increased alkali concentration and curing temperature, the morphology of geopolymers changed and formed fibrous and plate-like structures. The prevailing view is that a dense microstructure in the matrix enhances the fiber bridging capability, which is beneficial for improving deformation capacity and energy absorption [58].

Existing research generally suggests that the optimal oven heat curing conditions for EGC are primarily based on single-component precursors, with a recommendation of approximately 60 °C and a curing duration not exceeding 48 h. However, the effects of more commonly used two-component precursors and longer oven curing times on the performance of these materials have rarely been studied. Moreover, the fiber–matrix interface performance profoundly affects the ductility of EGC, and the mechanism of how curing conditions influence this microscopic behavior has not yet been fully explored. To further determine the optimal curing conditions for the mechanical properties of slag–fly ash-based high-ductility geopolymer concrete (HDGC), this study set three sealed curing temperatures (room temperature, 45 °C, and 60 °C) and four curing durations (1 day, 3 days, 5 days, and 7 days), while also considering two total curing ages (7 days and 28 days). This study explores the effects of curing conditions on the axial tensile and compressive mechanical properties of HDGC. Additionally, by examining the impact of curing temperatures on the microstructure of HDGC and the matrix–fiber interface, this research further investigates the fundamental mechanisms by which these curing conditions regulate the mechanical properties. Through this study, we aim to provide theoretical support for the design of low-carbon, high-performance concrete in terms of curing conditions, thus promoting innovation and development in sustainable building materials.

## 2. Materials and Methods

### 2.1. Materials

**Figure 1:**
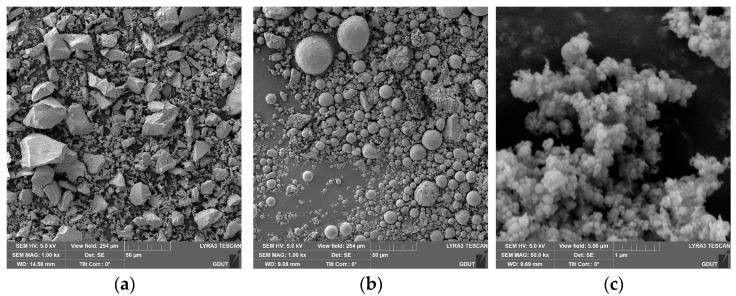
SEM of precursor materials: (**a**) GGBS, (**b**) FA, and (**c**) SF.

**Figure 2:**
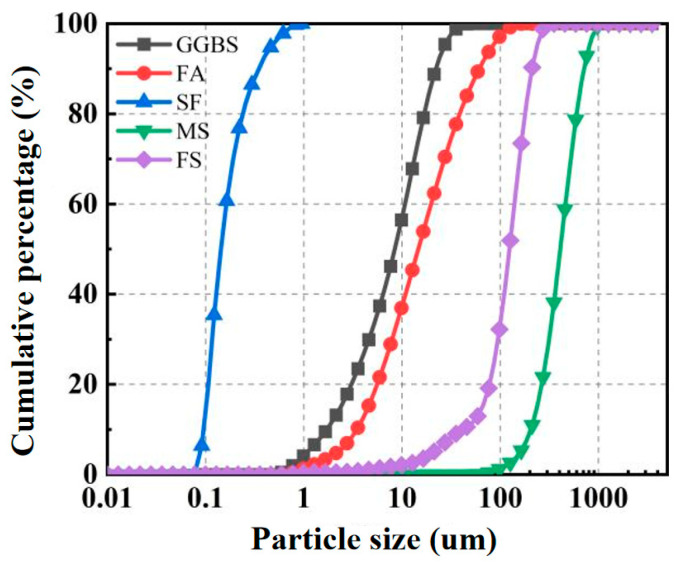
Cumulative particle size distribution curve.

The composition of HDGC studied in this paper includes precursors, aggregates, alkali activators, water, fibers, and admixtures. The precursors include Class F fly ash [59] (FA), Grade S105 ground granulated blast furnace slag (GGBS) [60], and silica fume (SF), all sourced from Longze Water Purification Materials Co., Ltd., in Gongyi City, China. The aggregates consist of quartz sand of two particle sizes: medium sand (MS, 0.15~0.40 mm) and fine sand (FS, 0.076~0.150 mm). The alkali activators are composed of a mixture of sodium hydroxide solution and sodium silicate solution, obtained from Xilong Scientific Co., Ltd., (Shantou, China) and Yourui Refractories Co., Ltd., (Jiaxing, China) in Jiashan County, China, respectively. The fibers used in HDGC are ultra-high molecular weight polyethylene fibers (UHMWPE fibers, hereafter referred to as PE fibers), sourced from Sovet Special Threads Co., Ltd., in Dongguan, China. The admixture is barium chloride dihydrate (abbreviated as BaCl_2_), used as a retarder to delay the initial setting time of HDGC, at a dosage of 1% by mass of the precursors, sourced from Xilong Scientific Co., Ltd. The SEM images of the precursor materials are shown in Figure 1. The particle size distribution curves of the precursor materials and the two aggregates are shown in Figure 2. From Figure 2, it can be seen that the particle size distribution of the components in HDGC is relatively uniform in this study. The physical and chemical parameters of all materials are listed in Table 1 and Table 2.

The HDGC mix used in this study is detailed in Table 3. The alkali activator consists of a 14 mol/L sodium hydroxide solution and a sodium silicate solution with a modulus of 2.25 (i.e., Na_2_O_2_ = 2.25), which are mixed to prepare a solution with a modulus of 1.5. Specifically, solid sodium hydroxide is first used to prepare a 14 mol/L sodium hydroxide solution, which is allowed to cool for 24 h. Once the solution has cooled to room temperature, it is mixed with the sodium silicate solution, stirred thoroughly, and allowed to stand until the temperature of the mixed solution returns to room temperature, ready for use.

**Figure 3:**
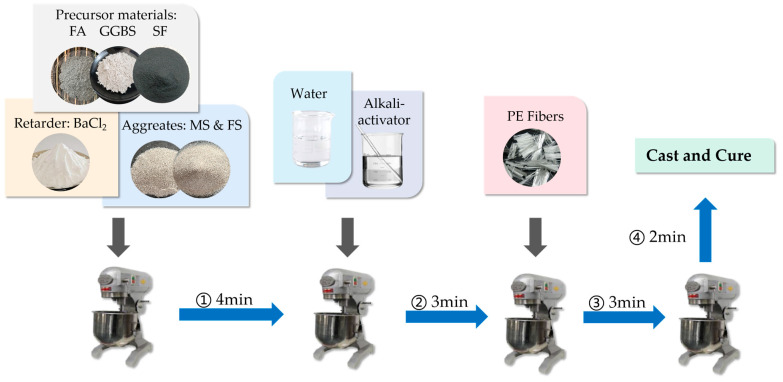
HDGC preparation procedure.

The specific process for preparing HDGC in this study is illustrated in Figure 3. First, the precursors, aggregates, and retarder are added to a planetary mixer and mixed for 4 min to ensure the dry materials are uniformly blended. In the second step, the cooled alkali activator is mixed with the required additional water and then added to the mixer, which is stirred for 3 min to complete the preparation of the HDGC matrix. In the final step, the pre-prepared 2 vol% PE fibers are evenly sprinkled into the mixer within 3 min while the mixer continues to operate. After the fibers are added, the mixer performs an additional 2 min of mixing to fully utilize the slurry’s lateral shear force to disperse the PE fibers, ensuring their even distribution throughout the HDGC matrix. Previous studies have shown that the uniformity of fiber distribution significantly affects both the workability and mechanical properties of fiber-reinforced concrete [61].

### 2.2. Curing Methods and Curing Time Design

**Figure 4:**
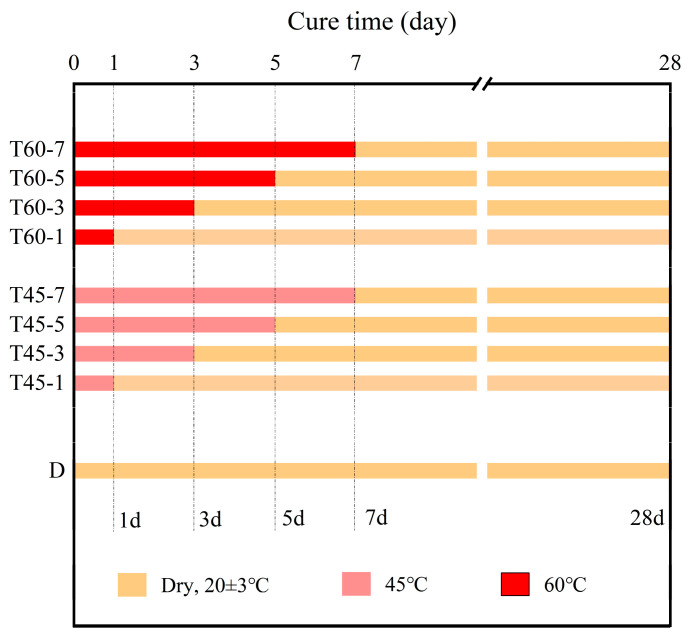
Test group setup.

The different curing conditions and durations for the specimens in this study constitute the primary focus of the research. The curing conditions are divided into four types: sealed indoor curing at room temperature (D-Y, room temperature 20 ± 3 °C), curing in a drying oven at 45 °C (T45-X-Y), and curing in a drying oven at 60 °C (T60-X-Y). Under the two different elevated temperature curing conditions, four curing durations of 1 day, 3 days, 5 days, and 7 days were designed, recorded as X = 1/3/5/7, to further investigate the impact of different temperatures and curing durations on the mechanical properties of HDGC. Additionally, specimens cured in the heated environment were removed from the drying oven after the corresponding curing time X and placed indoors to continue curing until 7 days and 28 days, recorded as Y = 7/28. In summary, the numbering of all experimental groups and their corresponding curing environments and durations in this study are shown in Figure 4.

### 2.3. Test Setup

#### 2.3.1. Compressive Test

**Figure 5:**
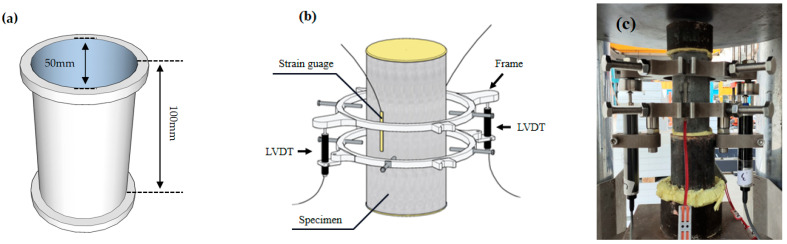
Compressive test setup: (**a**) the size of the mold, (**b**) schematic diagram of the test, (**c**) schematic diagram of the actual test.

The axial compression tests in this study were conducted following the ASTM-C469/C469M standards [62]. The tests utilized cylindrical specimens with a diameter of 50 mm and a height of 100 mm. Four symmetrically distributed strain gauges, each 30 mm in length, were attached around the middle of the specimens to measure axial and circumferential strains. Additionally, a pair of LVDTs (Linear Variable Differential Transformers) was installed in the axial position to measure the axial strain of the specimens. The tests were performed using a displacement control mode at a rate of 0.2 mm/min. The specific test setup and device configuration are illustrated in Figure 5.

#### 2.3.2. Tensile Test

**Figure 6:**
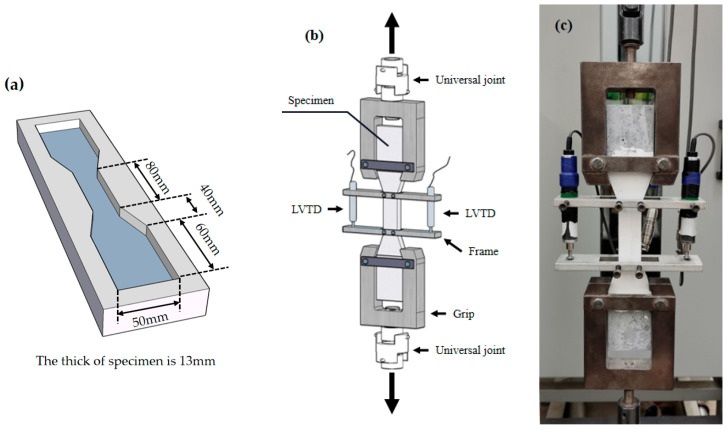
Tensile test setup: (**a**) the size of the mold, (**b**) schematic diagram of the test, (**c**) schematic diagram of the actual test.

In this study, HDGC dumbbell-shaped specimens were prepared according to JC/T2461-2018 [63]. The specimens were loaded in a tensile testing machine at a displacement rate of 0.5 mm/min. A pair of LVDTs were placed in the effective gauge length area in the middle of the dumbbell-shaped specimens to measure axial deformation. The specific specimen dimensions and test setup are shown in Figure 6.

#### 2.3.3. SEM and EDS Analysis

The scanning electron microscopy and energy-dispersive X-ray spectroscopy test were conducted using Pharos G2 scanning electron microscope, produced by PhenomScientific (Shanghai, China).

## 3. Results and Discussion

### 3.1. Compressive Test

#### 3.1.1. Stress–Strain Relationship of Uniaxial Compressive Test

**Figure 7:**
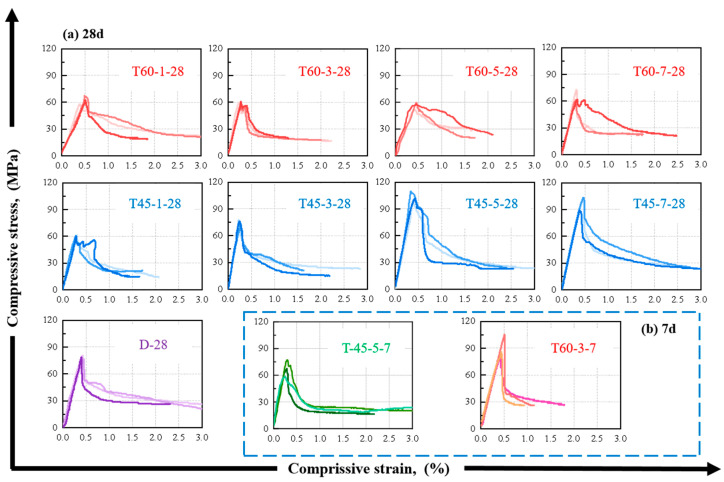
Compressive stress–strain curve of HDGC: (**a**) after aging to 28 d, (**b**) after aging to 7 d.

Figure 7a presents the axial compressive stress–strain curves of HDGC under different curing conditions after a 28-day curing age. The curves generally exhibit three stages: an elastic stage where stress increases steadily, a crack propagation stage where stress decreases post-cracking, and a failure stage where the slope of the stress–strain curve approaches zero. Notably, under the 45 °C curing condition, the longer the curing duration, the steeper and longer the elastic stage of the HDGC curve. In contrast, the results for the 60 °C curing condition showed the opposite trend; the elastic stage seemed to become gradually shorter. Additionally, the stress–strain curves of the specimens cured at room temperature (groups D) displayed a vertical line in the second stage, indicating that the specimens transition directly to the failure stage after cracking. However, the T45 and T60 series exhibited a descending branch post-cracking, known as the ductile failure descending section of concrete [2], with T45-5-28 being particularly prominent. Interestingly, Figure 7 reveals that the axial compressive strength of the T60 series is significantly lower than that of the specimens cured at room temperature, especially for T60-3-28.

Considering that T45-5-28 showed the high compressive strength and a relatively excellent ductility, T60-3-28 showed the opposite, and the compressive strength was even lower than that of D-28. Therefore, Figure 7b compares the axial compressive stress–strain curves of T45-5-7 and T60-3-7. The stress–strain curve of T45-5-28 appears to be the stress–strain curve following the development of compressive strength in T45-5-7. However, the stress–strain development of T60-3-28 and T60-3-7 differ significantly, with the former exhibiting a substantial strength reduction and the latter showing a brittle failure descending section post-cracking.

#### 3.1.2. Compressive Properties Parameters

**Figure 8:**
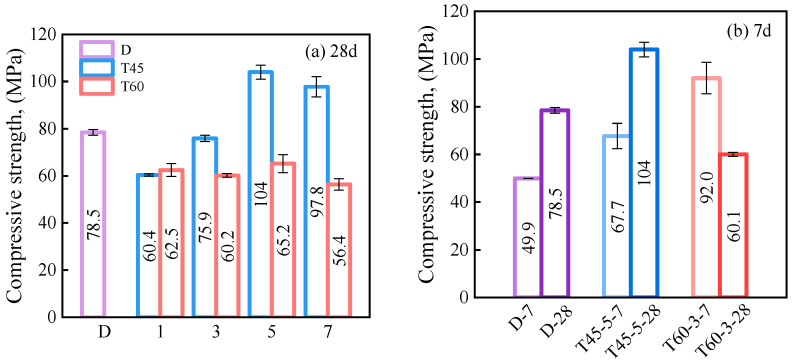
Compressive strength of HDGC: (**a**) after aging to 28 d, (**b**) after aging to 7 d.

Figure 8 illustrates the compressive strength of HDGC under different curing conditions, and Table 4 summarizes the compressive performance data of HDGC for each condition. In Figure 8a, it could be observed that only T45-5-28 and T45-7-28 exhibited a higher axial compressive strength than D-28, which showed a value of 78.5 MPa. This suggests that a curing temperature of 60 °C and curing times exceeding 3 days are not conducive to the 28-day strength development of the material. The highest compressive strength of 103 MPa was observed in T45-5-28, while the lowest compressive strength of 60.1 MPa was found in T60-3-28.

Figure 8b shows the 7-day compressive strength of certain test groups. Notably, the T45-5 and T60-3 series reveal that 60 °C significantly promotes the early strength development of HDGC. This observation is consistent with previous studies [34,64]. However, after 7 days of oven curing, the final strength at 28 days decreased by 34.7%. As other researchers have reported [65,66], prolonged oven curing can cause shrinkage and cracking of the precursor gel products, disrupting the original particle size distribution matrix and hindering later strength development. Additionally, Zhang [67] reported that at 60 °C or higher, zeolite crystals start to form in the matrix of alkali-activated fly ash-based materials, leading to changes in the pore structure of the matrix [68]. This explains the significant reduction in compressive strength for T60-3-28 after the removal from the thermal curing environment and subsequent aging. The performance differences demonstrated by the stress–strain curves of T60-3-28 and T45-5-28 in 3.1.1 are explained. Moreover, based on the test results of the T60 series in Figure 8a, it can be observed that the impact of oven curing time on compressive strength is not as direct as that of the oven curing temperature. Therefore, curing temperature plays a more decisive role in adjusting the compressive strength of HDGC.

### 3.2. Tensile Test

#### 3.2.1. Failure Mode

**Figure 9:**
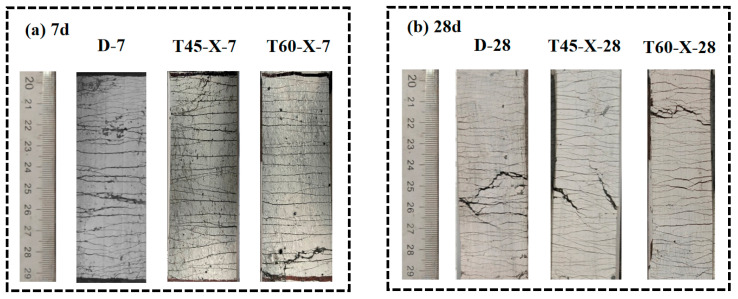
Failure mode of uniaxial tensile test: (**a**) after aging to 7 d, (**b**) after aging to 28 d.

Figure 9 presents the typical tensile failure modes of specimens at 7 days and 28 days under three different curing temperatures: D, T45, and T60. The images focus on the central 80 mm effective gauge length area. Visually, it can be observed that HDGC exhibited a characteristic multi-cracking failure mode under all three curing conditions. There is no significant difference in the number of cracks for specimens at the same age under different temperatures, but a noticeable change in the number of cracks is observed as the curing age increases from 7 days to 28 days. Kenda et al. [69] pointed out in their study on PE-fiber-reinforced composites that the development of $\sigma_{cu}^{n}$ (σ means strength) should meet the condition that $\sigma_{cu}^{n}>\sigma_{cu\;}^{i}(i=1,\;2,\;\cdot \cdot \cdot n-1)$ at that time, which means that a greater number of developed cracks indicates an improvement in the fiber bridging capacity.

#### 3.2.2. Tensile Stress–Strain Curves

**Figure 10:**
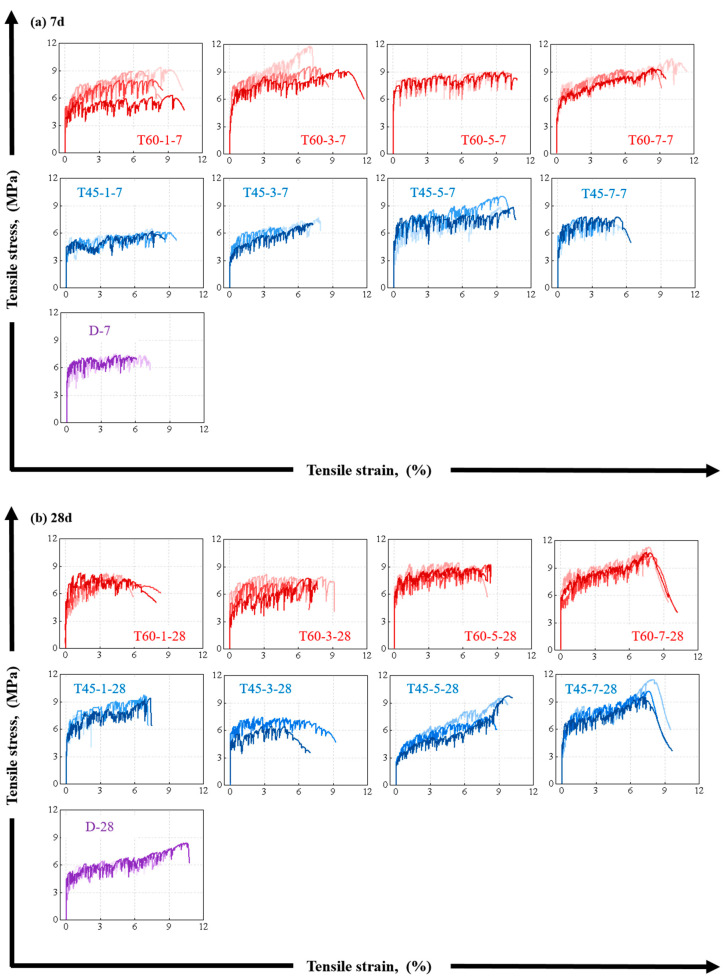
Stress–strain curve of uniaxial tensile test: (**a**) after aging to 7 d, (**b**) after aging to 28 d.

Figure 10 shows the tensile stress–strain curves of HDGC at 7 days and 28 days under different curing conditions. From Figure 10a, it can be seen that all test groups exhibited three stages in tensile stress development: elastic development, pseudo-strain-hardening, and failure softening. Notably, within the 7-day curing period, a curing temperature of 60 °C is more conducive to the development of tensile strain during the pseudo-strain-hardening stage of HDGC, and the highest ultimate tensile strain is visually observed in T60-7-7. Additionally, the pseudo-strain-hardening stage is significantly longer in the T60 series compared to the T45 series. When the curing age reached 28 days, the pseudo-strain-hardening stage shortened in some test groups, such as T45-1-28, T45-3-28, T60-1-28, and T60-5-28. Specifically, the pseudo-strain-hardening stage extended in T45-7-28, with a significant increase in tensile strength during this stage, following the same pattern as seen in the D-series test group.

As shown in Figure 10, the peak tensile strength of pseudo-strain-hardening materials like HDGC is strongly associated with their stiffness and fiber bridging capability. Stiffness is reflected in the slope of the stress–strain curve during the elastic stage, while the fiber bridging capability is crucial for achieving pseudo-strain-hardening in HDGC. Notably, the fiber bridging capability alone cannot directly determine the final crack development; the fracture toughness of the matrix must also be considered [58,69].

#### 3.2.3. Tensile Properties Parameters

**Figure 11:**
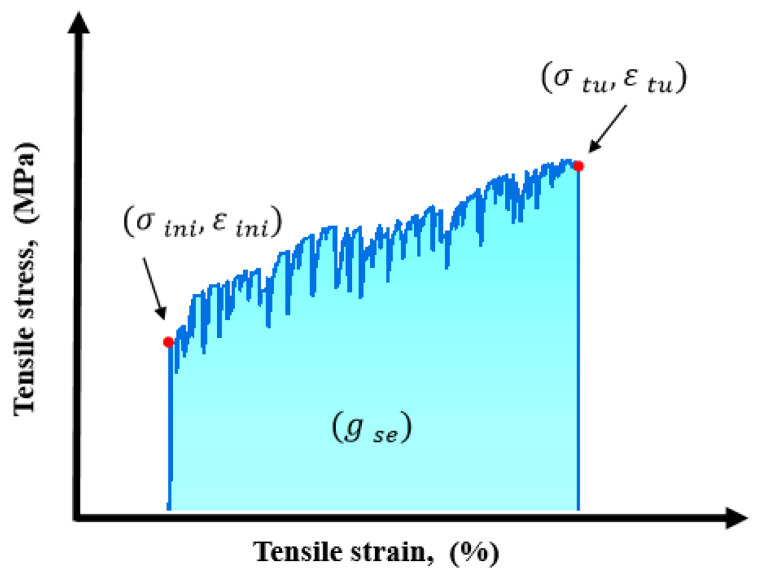
Characteristic parameters of direct tensile test for HDGC.

Figure 11 shows the typical tensile stress–strain curves of pseudo-strain-hardening materials, which include the following tensile performance characteristic parameters: initial crack stress ($\sigma_{ini}$), initial crack strain ($\varepsilon_{ini}$), tensile strength ($\sigma_{tu}$), ultimate tensile strain ($\varepsilon_{tu}$), and strain energy density ($g_{se}$). The strain energy density is obtained by integrating the area under the curve from the origin to the point corresponding to the ultimate tensile strength; the specific calculation formula refers to Equation (1).

(1) $$g_{se}=\int_{0}^{\varepsilon_{tu}}\sigma (\varepsilon_{t})d\varepsilon_{t},\;0\leq \varepsilon_{t}\leq \varepsilon_{tu}$$

**Figure 12:**
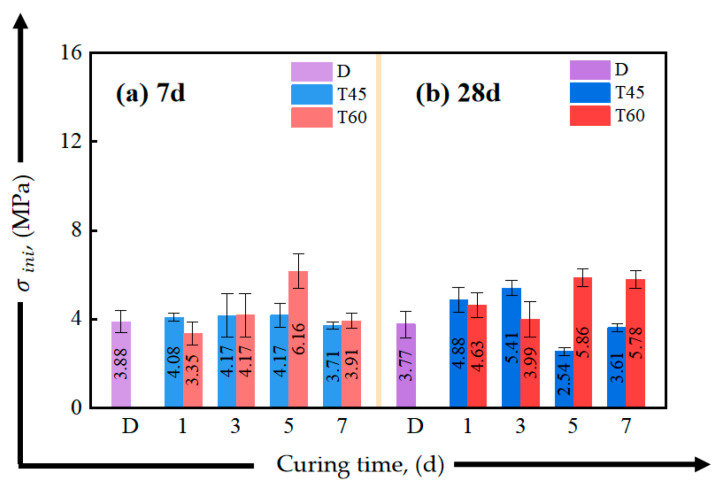
Effect of curing time on initial cracking strength of direct tensile test: (**a**) after aging to 7 d, (**b**) after aging to 28 d.

**Figure 13:**
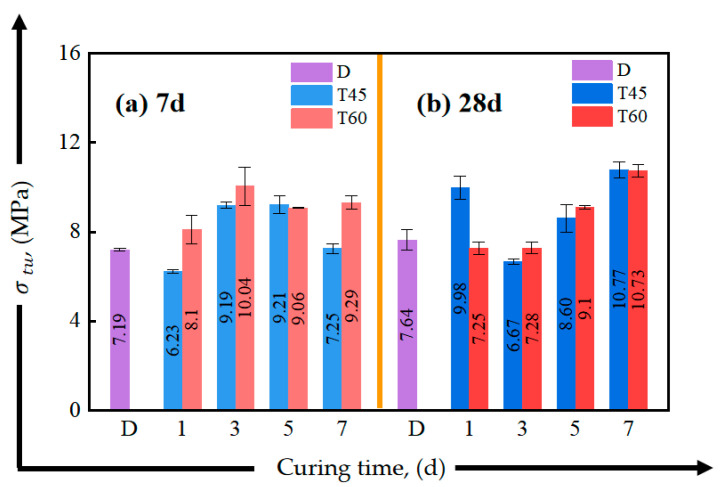
Effect of curing time on peak tensile strength of direct tensile test: (**a**) after aging to 7 d, (**b**) after aging to 28 d.

Figure 12 and Figure 13 present the effects of different curing conditions on the tensile properties of HDGC at 7-day and 28-day curing ages. Table 5 summarizes the corresponding tensile performance parameters. From Figure 12, it can be seen that curing at 45 °C has almost no impact on the 7-day initial crack strength, which remains nearly the same as the D series. In the T60 series, only a 5-day curing duration significantly improved the initial crack strength, with the lowest initial crack strength observed in T60-1-7. At the 28-day curing duration, the initial crack strength of T45-5-28, T45-7-28, T60-3-28, and T60-5-28 failed to increase. Combining this with the similar findings of the D series, it can be inferred that extending the curing duration does not necessarily enhance the initial crack strength; the early curing environment may have a greater influence on the tensile stiffness of HDGC.

From Figure 13, it can be observed that the peak tensile strength of each group has increased compared to the initial cracking strength. We can specifically observe from Table 5 that the increases in the peak tensile strength relative to the initial cracking strength are 142% and 239% for T60-1-7 and T45-5-28, respectively, corresponding to $\sigma_{tu}$/$\sigma_{ini}$ values of 2.42 and 3.39. These are the largest values in their respective age series. This finding is consistent with the results discussed in the stress–strain curves. Notably, T60-1-7 and T45-5-28 exhibited the greatest increase in tensile stress during the pseudo-strain-hardening phase, correspond to the groups with the lowest initial crack strength in the 7-day and 28-day series, respectively. Considering the mechanism analysis in Section 3.1.2, which suggests that oven curing conditions can induce zeolite formation in the matrix, it can be concluded that zeolite affected the matrix stiffness by altering the pore structure. Specific oven curing conditions could enhance the fiber bridging capacity, thereby improving the material’s strain-hardening capability and resulting in a more significant stress increase [58,69].

**Figure 14:**
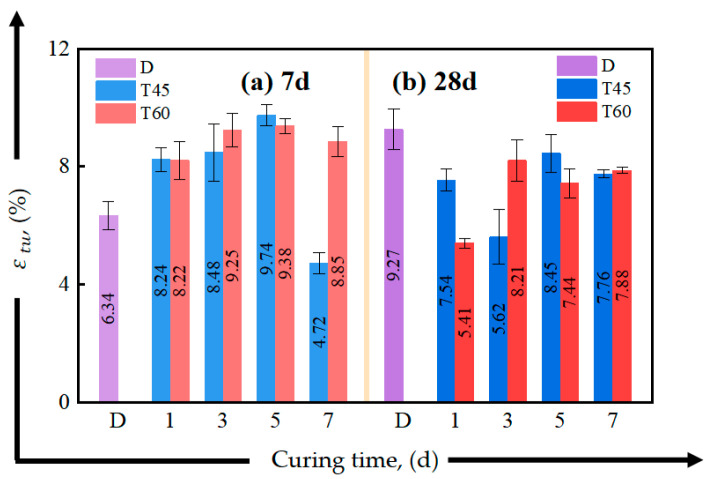
Effect of curing time on peak tensile strain of direct tensile test: (**a**) after aging to 7 d, (**b**) after aging to 28 d.

**Figure 15:**
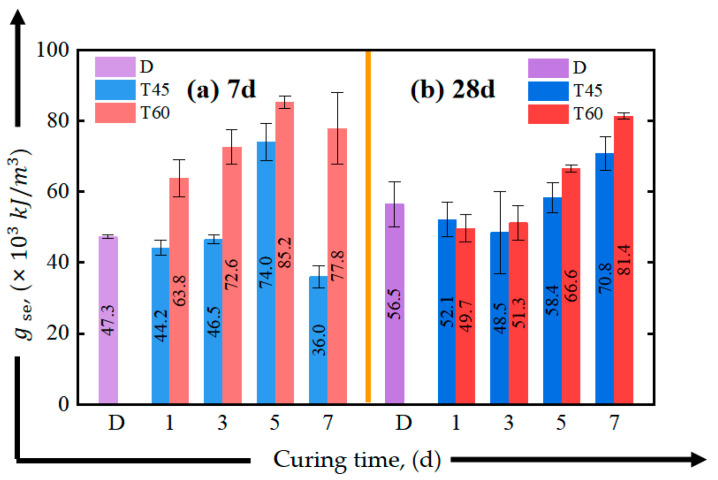
Effect of curing time on strain energy density of direct tensile test: (**a**) after aging to 7 d, (**b**) after aging to 28 d.

The initial cracking in concrete materials occurs early and randomly [70,71,72], so this section will not discuss the influence of curing conditions on the material’s initial cracking stress and strain. As shown in Figure 14 and Figure 15, oven curing temperatures of 45 °C and 60 °C were beneficial for the early (7-day) ductility development of the material, especially for T45-5-7 and T60-3-7, and the peak strain increased by 53.6% and 45.9% compared to D-7, respectively. Additionally, oven curing for 5 days or more helped to enhance the tensile strain energy density of the HDGC, as shown in Figure 15. Both T60-5 and T60-7 exhibited top-tier tensile energy absorption capacity at both the 7-day and 28-day curing ages. The highest value was observed in T60-5-7 at 85.2 kJ/m^3^, which is 1.8 times that of D-7.

### 3.3. Microstructure Analysis

#### 3.3.1. The Influence of Curing Temperature on the Development of the Matrix

**Figure 16:**
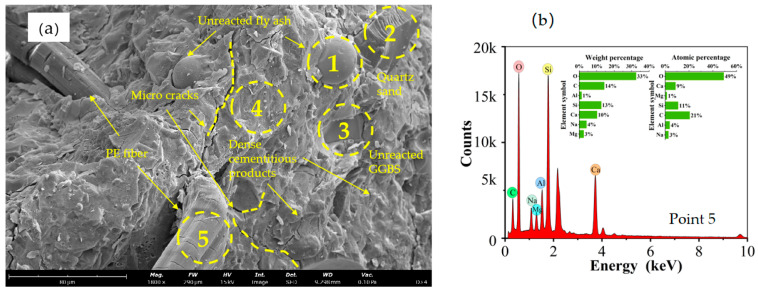
Microstructure analysis of Group D: (**a**) SEM image, (**b**) EDS analysis of Point 5.

Figure 16 presents the SEM micrographs and EDS analysis results for HDGC under different curing conditions. Given the significant improvements in tensile strength and energy absorption for T45 and T60 after 7 days of curing, samples were taken from the fracture surfaces of T45-7-28 and T60-7-28 tensile specimens. Taking the microstructure of D-28 shown in Figure 16 as an example, Points 1 and 3 indicate unreacted fly ash and slag, Point 2 indicates quartz sand, Point 4 indicates gel products, and the elongated objects are PE fibers. EDS analysis of the PE fibers (Point 5) is shown in Figure 14b. The Ca/Si ratio is 0.818, and the Al/Si ratio is 0.363. Based on atomic percentage data [73], the attached material on the fibers is identified as C-(N)-A-S-H gel. Notably, the strong bonding ability of C-(N)-A-S-H gel contributes to enhancing the adhesion of PE fibers within the HDGC matrix.

**Figure 17:**
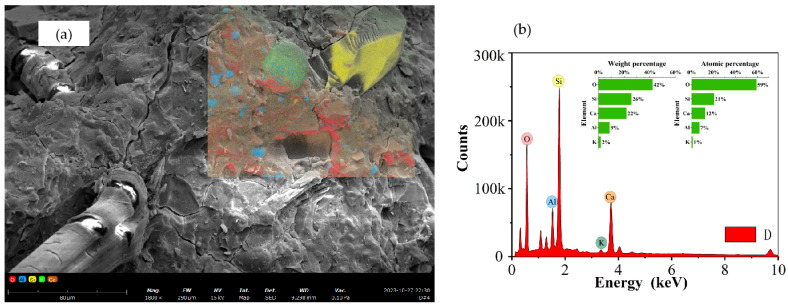
EDS mapping results of D-28: (**a**) surface scan schematic, (**b**) EDS results.

**Figure 18:**
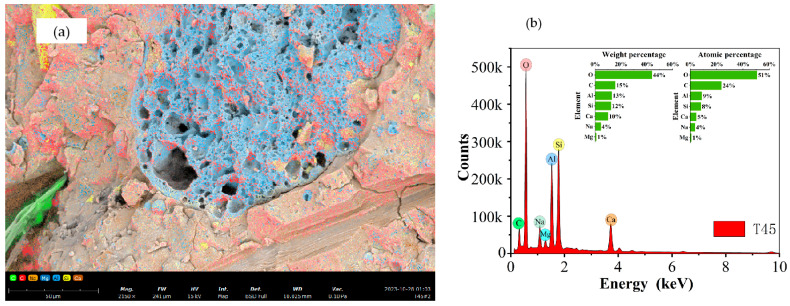
EDS mapping results of T45-7-28: (**a**) surface scan schematic, (**b**) EDS results.

**Figure 19:**
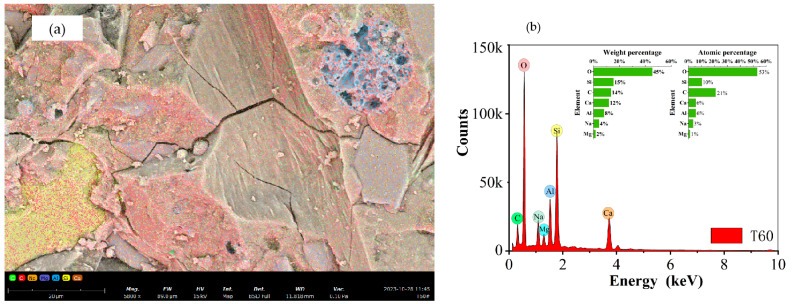
EDS mapping results of T60-7-28: (**a**) surface scan schematic, (**b**) EDS results.

**Figure 20:**
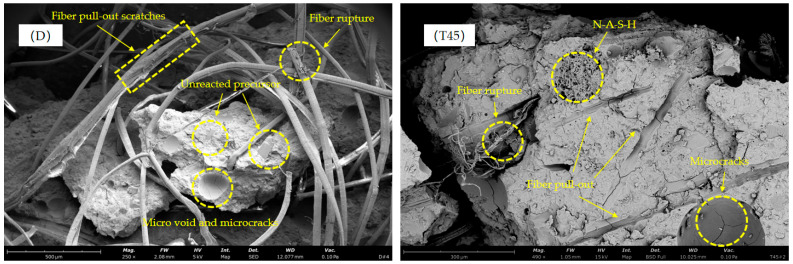

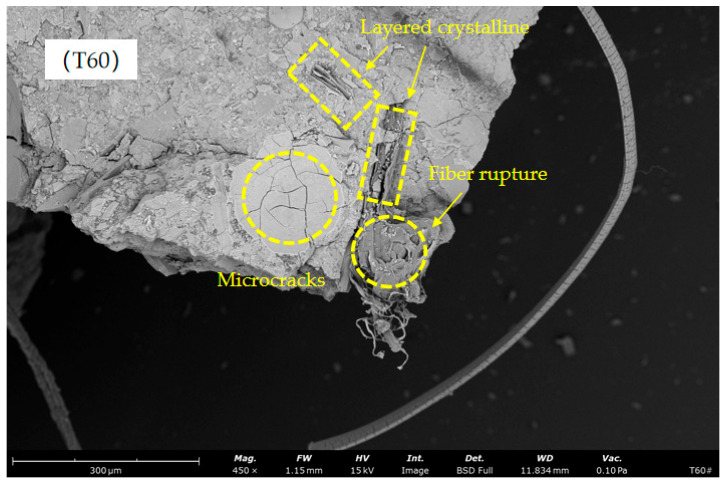
SEM images of the matrix and fibers: (D) D-28, (T45) T45-7-28, (T60) T60-7-28.

Based on the SEM micrographs and EDS surface scan results in Figure 17, Figure 18, Figure 19 and Figure 20, the Ca/Si ratio of the matrix is approximately 0.6, while the Al/Si ratio increases with higher curing temperatures. This is because elevated temperatures accelerate the dissolution of alumina in the precursor, promoting the precursor’s reaction. Additionally, temperature changes can alter the morphology of fly ash, leading to the formation of multilayered, plate-like gel structures within the matrix, as shown in Figure 20 (T60). Overall, at the same scale, the microstructure of the materials under all three curing conditions appears to be well-developed and dense.

#### 3.3.2. The Influence of Curing Temperature on the Bonding between Matrix and Fiber

Figure 20 shows the effect of different curing temperatures on the bonding between the HDGC matrix and fibers. The SEM images reveal some undissolved precursors and pores formed due to moisture evaporation during mixing or after the matrix has set. It is evident that these undissolved precursors and the surrounding pores have microcracks, which directly affect the density of the matrix and the bonding strength between the fibers and the matrix. As observed in Section 3.3.1, a higher Al/Si ratio due to oven curing indicates an improved precursor reaction, enhancing the matrix’s density and thus the fiber bridging performance. This confirms the mechanisms discussed in Section 3.2.3, where specific oven curing conditions were found to enhance the fiber bridging ability and improve the material’s pseudo-strain-hardening effects.

#### 3.3.3. The Influence of Curing Temperature on the Fiber Failure Mode

**Figure 21:**
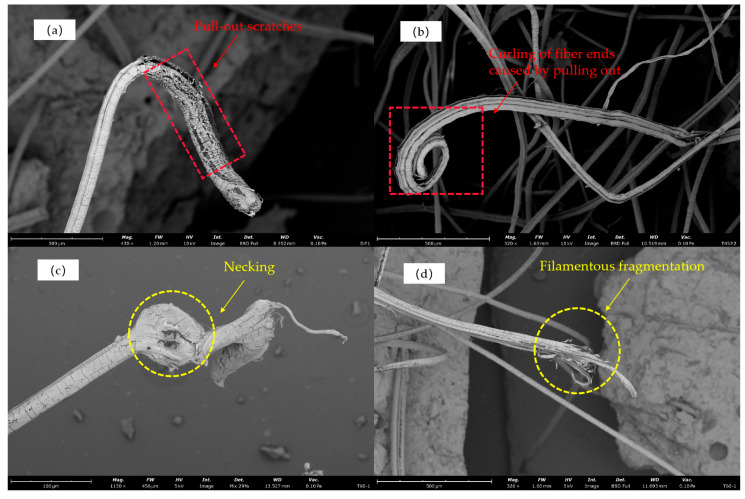
Fiber failure mode: (**a**) pullout scratches, (**b**) curling of fiber ends, (**c**) necking, (**d**) filamentous fragmentation.

The specific failure modes of fibers are illustrated in Figure 21. The morphology of fiber tensile failure can be categorized into two types: fiber pullout and fiber rupture.

Fiber Pullout: Under microscopic examination, a dense network of cracks is observed on the fiber surface, or there is surface delamination. The fibers are elongated and exhibit flattened or curled modes; specifically, refer to Figure 21a,b.

Fiber Rupture: This mode is characterized by fine, randomly tangled filaments, fibers fracturing into multiple strands, or local yielding reaching the extreme limit, leading to necking and rupture at the ends; specifically, refer to Figure 21c,d.

**Figure 22:**
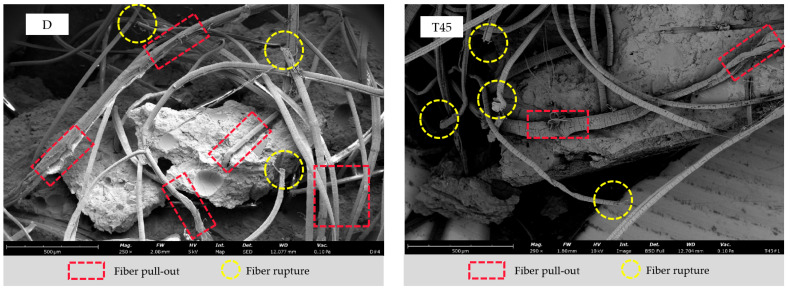

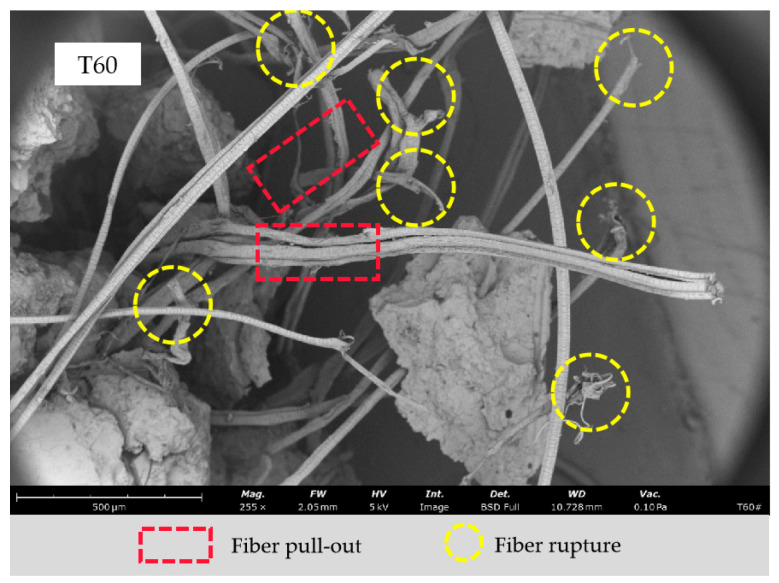
The effect of curing temperature on fiber failure modes: (D) D-28, (T45) T45-7-28, (T60) T60-7-28.

From Figure 22, it can be observed how the three curing temperatures affect the fiber failure modes. At the same observation scale, fiber pullout failure is more commonly seen in the D group with room temperature curing. This is manifested by noticeable damage on the fiber surface due to friction with the matrix, while the fiber ends remain intact. In contrast, the T45 and T60 oven-cured samples show a richer variety of fiber rupture failure modes, including fiber end necking and splitting into fine strands. Specifically, oven curing enhanced the adhesion between the fibers and the matrix, leading to a closer bond and an improved fiber bridging effect. On the other hand, this also mitigated the negative impact of low-modulus fibers on the overall modulus of HDGC, as evidenced by the increased axial compressive modulus in the T45 and T60 series, thereby increasing the rigidity of HDGC.

## 4. Conclusions

In this study, three curing temperatures (room temperature, 45 °C, and 60 °C), four curing durations (1 day, 3 days, 5 days, and 7 days), and final curing ages (7 days and 28 days) were investigated to study their effects on the axial compressive and tensile properties of HDGC. The mechanisms behind the impact of curing conditions on the mechanical properties of HDGC were also explored, by analyzing microstructural changes under different curing temperatures. The conclusions are as follows:

(1) Curing at 45 °C and 60 °C allows HDGC to enter a ductile failure stage after reaching compressive strength, unlike the room temperature-cured samples, which show splitting cracks and immediate failure. A 5-day heat curing at 45 °C maximizes the compressive strength of HDGC and extends its ductile failure phase. Therefore, 45 °C is recommended as the optimal oven curing temperature to improve HDGC’s compressive performance.

(2) The 28-day compressive strength of HDGC cured at 60 °C is significantly lower than at 7 days. The formation of zeolite crystals from the slag and fly ash precursors at this temperature significantly affects the pore structure of the matrix, leading to a decrease in matrix strength during long-term curing and a reduction in compressive strength over time.

(3) T60-3-7 and T45-5-28 exhibited significant tensile strain hardening, yet had the lowest initial tensile cracking strength. Specific oven curing conditions can reduce the initial cracking toughness of the matrix while enhancing the fiber bridging capability, thereby increasing the degree of strain hardening in HDGC according to the criteria for pseudo-strain-hardening materials. This also helps to improve the energy dissipation capacity of the material.

(4) Higher curing temperatures promote the full reaction of the precursors, increasing the bond strength between the matrix and fibers. This enhances fiber bridging capability while reducing the negative impact of low-modulus PE fibers on the overall stiffness of the material. Oven curing improved the compressive elastic modulus of HDGC.

(5) Considering the overall effects of curing conditions on HDGC’s mechanical properties, this study recommends 45 °C as the optimal curing temperature. For long-term mechanical performance, which is crucial for concrete materials, a heat curing duration of 5 to 7 days is the better choice.

## Figures and Tables

**Table 1:** Property parameters of precursor materials.

Property Parameters.	CaO	SiO_2_	Al_2_O_3_	SO_3_	Fe_2_O_3_	MgO	TiO_2_	Other	Loss on Ignition (%)	Density (g/cm^3^)
	wt %									
GGBS	34.0	34.5	17.7	1.64	1.03	6.01	/	5.12	0.840	2.90
FA	4.01	54.8	31.2	2.20	4.16	1.01	1.13	2.37	4.60	2.30
SF	/	94.7	/	0.20	/	/	/	5.10	3.00	0.25

**Table 2:** Physical and mechanical properties of PE fibers.

Diameter *d_f_* (μm)	Length *L_f_* (mm)	Strength (MPa)	Elastic Modulus (GPa)	Elongation (%)	Density (g/cm^3^)	Melting Temperature (°C)
20.0	6/12/18	>3000	>110	3.5–4	0.970	135–145

**Table 3:** Mix ratio of HDGC (unit: kg/m^3^).

GGBS	FA	SF	MS	FS	NaOH Solution	Sodium Silicate Solution	Extra Water	PE Fiber	BaCl_2_
924	280	76.0	68.0	385	105	439	78.8	21.9	12.7

**Table 4:** Compressive properties parameters under different curing conditions.

Specimen	Compressive Strength (MPa)	Peak Compressive Strain (%)	Elastic Modulus (GPa)
D-7	49.9 (0.9)	0.005 (0.001)	14.4 (0.9)
D-28	78.5 (1.5)	0.004 (0.002)	21.5 (1.8)
T45-1-28	60.4 (0.5)	0.003 (0.001)	22.9 (2.7)
T45-3-28	75.9 (1.3)	0.002 (0.001)	34.1 (0.7)
T45-5-28	104 (2.9)	0.004 (0.003)	28.1 (1.4)
T45-7-28	97.8 (4.3)	0.004 (0.001)	25.0 (0.5)
T60-1-28	62.5 (2.8)	0.005 (0.002)	13.1 (0.7)
T60-3-28	60.2 (0.8)	0.003 (0.001)	27.8 (3.4)
T60-5-28	65.2 (3.8)	0.003 (0.002)	22.3 (0.6)
T60-7-28	56.4 (2.4)	0.004 (0.001)	15.3 (1.2)
T45-5-7	67.7 (5.3)	0.003 (0.001)	34.3 (5.5)
T60-3-7	91.979 (6.575)	0.005 (0.001)	22.1 (1.1)

**Table 5:** Tensile properties parameters under different curing conditions.

Specimen	$\sigma_{ini}$ (MPa)	$\varepsilon_{ini}$ (%)	$\sigma_{tu}$ (MPa)	$\varepsilon_{tu}$ (%)	$g_{se}$$\;(\times 10^{3}$ kJ/m^3^)	$\sigma_{tu}$ $/\sigma_{ini}$
D-7	3.88 (0.49)	0.021 (0.006)	7.19 (0.08)	6.34 (0.47)	47.3 (0.5)	1.86
D-28	3.77 (0.60)	0.022 (0.004)	7.64 (0.44)	9.27 (0.69)	56.6 (6.3)	2.03
T45-1-7	4.08 (0.17)	0.034 (0.003)	6.23 (0.09)	8.24 (0.41)	44.2 (2.1)	1.53
T45-3-7	4.17 (0.98)	0.031 (0.007)	9.19 (0.15)	8.48 (0.98)	46.5 (1.2)	2.21
T45-5-7	4.17 (0.52)	0.021 (0.005)	9.21 (0.41)	9.74 (0.36)	74.0 (5.2)	2.21
T45-7-7	3.71 (0.17)	0.017 (0.003)	7.25 (0.21)	4.71 (0.36)	36.0 (3.1)	1.95
T60-1-7	3.35 (0.50)	0.018 (0.004)	8.09 (0.65)	8.22 (0.64)	63.8 (5.2)	2.42
T60-3-7	4.17 (0.97)	0.032 (0.007)	10.0 (0.9)	9.25 (0.57)	72.6 (5.0)	2.42
T60-5-7	6.16 (0.77)	0.043 (0.015)	9.06 (0.02)	9.38 (0.26)	85.1 (1.8)	1.47
T60-7-7	3.91 (0.34)	0.023 (0.004)	9.29 (0.30)	8.85 (0.50)	77.8 (10.1)	2.37
T45-1-28	4.87 (0.55)	0.025 (0.002)	9.99 (0.51)	7.54 (0.37)	52.1 (4.8)	2.05
T45-3-28	5.40 (0.34)	0.030 (0.004)	6.67 (0.14)	5.62 (0.92)	48.5 (11.6)	1.23
T45-5-28	2.54 (0.19)	0.022 (0.002)	8.60 (0.62)	8.45 (0.63)	58.4 (4.2)	3.39
T45-7-28	3.61 (0.19)	0.018 (0.001)	10.8 (0.4)	7.76 (0.14)	70.8 (4.8)	2.98
T60-1-28	4.63 (0.56)	0.025 (0.005)	7.25 (0.28)	5.41 (0.16)	49.7 (3.8)	1.57
T60-3-28	3.99 (0.79)	0.030 (0.002)	7.28 (0.26)	8.21 (0.70)	51.3 (4.9)	1.82
T60-5-28	5.86 (0.42)	0.029 (0.003)	9.10 (0.09)	7.44 (0.49)	66.6 (0.9)	1.55
T60-7-28	5.78 (0.40)	0.028 (0.003)	10.7 (0.3)	7.88 (0.09)	81.4 (0.92)	1.86

## Data Availability

The data presented in this study are available on request from the corresponding author. The data are not publicly available due to confidentiality issues.
